# Naphthylimide Chemosensor Based on Anion−π Interactions: A Promising Tool for Environmental Monitoring

**DOI:** 10.1021/acsomega.5c02699

**Published:** 2025-07-10

**Authors:** Janail Rodrigues da Silva, Vinicius Flores da Silva, Willyan Farias Oliveira, Ricardo Oliveira Freire, João Honorato de Araujo-Neto, Izilda A. Bagatin

**Affiliations:** † Instituto de Ciências Ambientais, Químicas e Farmacêuticas—Depto de Química, Laboratório de Química de Calixarenos, Espectroscopia Molecular e Catálise, 28105 Universidade Federal de São Paulo, Rua Prof. Arthur Riedel, 275, CEP 09972-270 Diadema, SP, Brazil; ‡ Pople Computational Chemistry Laboratory, Department of Chemistry, 74391 UFS, 49107-230 São Cristóvão, SE, Brazil; § Department of Fundamental Chemistry, Institute of Chemistry, 28133 University of São Paulo, 05508-000 São Paulo, SP, Brazil

## Abstract

In the domain of environmental monitoring, chemosensors play a pivotal role, particularly in the detection of ions such as fluoride. Although this element is beneficial in controlled doses, it menaces human health because it can attack tooth enamel in high concentrations. Our research group has developed a selective chemosensor for fluoride ions based on a naphthylimide derivative. This study employed a multifaceted approach involving ultraviolet-visible (UV–vis), fluorescence, and NMR spectroscopic techniques, complemented by theoretical calculations, to elucidate the interaction. The employment of UV–vis methods revealed a *K*_11_ value of 2.0 × 10^4^ mol dm^–3^ for the chemosensor, indicating its notable stability toward fluoride. A critical aspect of the probe’s functionality is its anion−π interaction mechanism with the analyte, a finding substantiated by ^1^H NMR titrations. These titrations demonstrated that the primary interaction occurred with the aromatic rings of the benzene moiety rather than with the N–H group. Theoretical calculations corroborate these findings and highlight the viability of anion−π interactions. This chemosensor boasts numerous advantages, including cost-effectiveness, ease of use, and high selectivity for fluoride. Consequently, they have emerged as promising tools for environmental and analytical applications.

## Graphical abstract

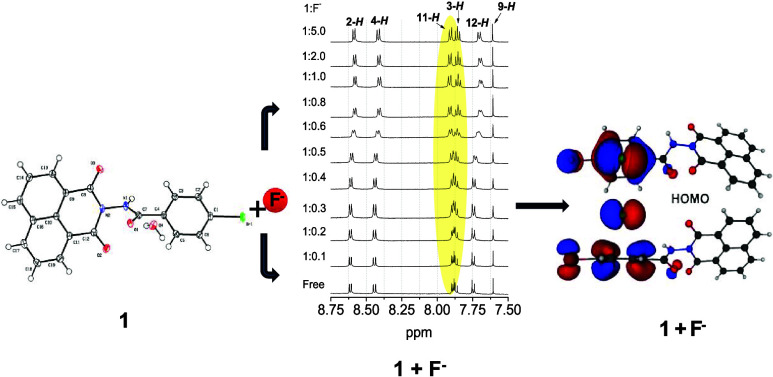

## 1 Introduction

A sensor is an instrument that can identify and quantify matter or energy.^1^ A chemosensor is defined as a molecule or supramolecule that functions as a sensor, thereby enabling the identification of analytes^2^ or measurement of compounds in a specific medium, whether this is a solution or gas.^3^ The ability of chemosensors to analyze and quantify hazardous species has been crucial to environmental protection, making them indispensable tools in everyday life. In this context, chemosensors have been developed as low-cost alternatives to solve these environmental problems. However, with the advent of chemosensors, significant challenges in searching for practical alternatives remain, worth highlighting in this discussion.

Fluoride ions, for instance, benefit dental health when administered in moderate doses. However, when present in excess, they can compromise the integrity of tooth enamel, leading to hypomineralization or porosity, which in turn can result in dental fluorosis.^4^

Naphthylimides represent a class of organic compounds that are of significant importance. They are employed in various applications, including developing organic light-emitting diodes (OLEDs),^5–9^ semiconductor materials,^10–13^ solar cells,^14–17^ and chemosensors.^18–22^ Their ability to emit light when excited by a suitable light source renders them valuable tools in creating chemosensors for various applications, including antiviral research^23–25^ and environmental monitoring.^26–28^

Fluoride ions in water can be identified using various techniques including electrochemical methods and staining. However, both methods are subject to limitations due to the presence of interfering ions in water. In response to the need for new alternatives, recent research has focused on investigating molecules that are capable of readily interacting with anions via hydrogen bonding. This approach aims to combine sensitivity, selectivity, and sustainability.

This area has recently attracted considerable interest. For instance, Qu et al.^29^ developed a polymeric chemosensor based on naphthylimide for fluoride ions. This sensor exhibits spectral and intensity changes in the presence of fluoride, which can be observed by fluorescence or colorimetry. It can detect fluoride concentrations as low as 1 × 10^–6^ mol dm^–3^, with changes in the visible spectrum.

Xiao et al.^30^ presented another example of a colorimetric and fluorometric chemosensor for identifying fluoride ions modified with 1,8-naphthylimide: the presence of fluoride results in a blue solid coloration, with a limit of detection (LOD) of 8.06 × 10^–7^ mol dm^–3^.

Furthermore, Wu et al.^31^ presented a colorimetric and fluorescent probe based on 1,8-naphthyllimide, 4-(2,2-dichloroacetamide)-*N*-butylnaphthyllimide (CNA), which was designed for the detection of fluoride anions in CH_3_CN. A notable change from colorless to yellow of the CNA solution was observed with the naked eye in the presence of F^–^ among the other anions, accompanied by a reduction in the fluorescence intensity. The detection limits of CNA for F^–^ were 0.52 and 1.41 μM, as determined by the fluorescence and absorption spectra, respectively.

Naphthylimides were established by Sun et al.,^32^ who developed a fluorescent probe for detecting dithiothreitol (DTT), a reducing agent widely used in biology, biochemistry, and biomedicine. The probe developed showed satisfactory selectivity and a detection limit of 1.4 × 10^–7^ mol dm^–3^.

In this context, this study aims to advance the field of naphthylimide-based chemosensors by introducing a novel structural modification of 1,8-naphthalimide to select potentially toxic species. This research contributes to ongoing efforts to develop cost-effective and highly sensitive sensors for environmental monitoring and analytical applications.

## 2 Experimental Section

### 2.1 Materials and Methods

A Schlenk flask and standard glassware were used for the synthesis, which was performed under an inert gas atmosphere (N_2_). All solvents were of PA-ACS grade. The reagents were of PA grade and used without purification, and were purchased from Sigma-Aldrich or Merck. The ligand synthesis was accompanied by thin layer chromatography (TLC) on Merck silica gel 60 (0.25 mm thick).

Nuclear magnetic resonance (^1^H NMR) spectra were recorded in deuterated chloroform (CDCl_3_), DMSO, or acetonitrile-*d*_3_ (CD_3_CN) (“100%”, 99.96 atom % D, Aldrich) solvent (0.6 mL) on a Bruker Avance III Ultrashield 300 spectrometer (300 MHz) and a Bruker Ascend 500 spectrometer (500 MHz). The ^1^H NMR data were referenced to residual CHCl_3_ (7.27 ppm) or CH_3_CN (1.94 ppm) and 2D NMR [^1^H–^1^H correlation spectroscopy (COSY), ^1^H–^13^C heteronuclear single quantum coherence spectroscopy (HSQC), and ^1^H–^13^C heteronuclear multiple bond correlation spectroscopy (HMBC)] referenced to DMSO (2.54 ppm) in a solvent mixture (CDCl_3_/DMSO, 0.5:0.1 v/v) performed with gradient.

IR spectra were recorded on dry KBr pellets using a FTIR Shimadzu Prestige-21 instrument.

Electronic spectra were recorded at a concentration of 2 × 10^–5^ mol dm^–3^ in acetonitrile by using a Hewlett–Packard model 8453 diode array spectrophotometer (D2 and W lamps) with a 1 cm double-sided quartz cuvette.

Fluorescence spectra were obtained using a Fluorolog-3 HORIBA FL3C-22 spectrophotometer with a 450 W lamp and a 5 nm (*E*M) and 1 nm (*E*XC) slit using a 1 cm four-sided quartz cuvette and a concentration of 1 × 10^–6^ mol dm^–3^ in deaerated acetonitrile.

### 2.2 Determination of Acid Dissociation and Association Constants

The experiments were carried out in a solution containing 1 × 10^–5^ mol dm^–3^ of *N*-4-bromobenzamide-1,8-naphthylimide **1** in an acetonitrile (CH_3_CN) solution. Then, 3 mL of this solution of ligand **1** was placed in a cuvette, and the first spectrum (free ligand) was recorded. Next, titration was started by adding appropriate μL of sodium hydroxide (NaOH) solution (1 × 10^–2^ mol dm^–3^) and adjusting the volumes corresponding to each pH to determine the p*K*_a_ of this molecule. Because the added volumes (μL) were infinitely smaller than the total volume of the cuvette (3 mL), there was no dilution effect or any considerable volume change, as observed even by the baseline of the UV–vis spectrum. Table S1 lists the NaOH units (μL) added to the solution in the cuvette, corresponding to the observed pH (apparent).

### 2.3 Analysis of the Molecular Interaction between *N*-4-Bromobenzamide-1,8-naphthylimide with F^–^ by ^1^H NMR

This experiment was carried out using deuterated acetonitrile (CD_3_CN), starting with a solution of 1.0 × 10^–3^ mol dm^–3^ of ligand **1** in an NMR tube and adding F^–^ ions (in a solution of 6.8 × 10^–1^ mol dm^–3^ dissolved in D_2_O) into the same tube, in the proportion of 1:0.1, 1:0.2, 1:0.3, 1:0.4, 1:0.5, 1:0.6, 1:0.8, 1:1.0, 1:2.0, and 1:5.0.

### 2.4 Structure Determination by Single-Crystal X-ray Diffractometry

Single crystals of C_19_H_13_BrN_2_O_4_ were obtained by slow evaporation of a mixture of solvents in the proportions 0.6:0.4 v/v (CHCl_3_/CH_3_CN). Single-crystal X-ray diffraction analyses were carried out at a collection temperature of 100 K using a Rigaku Synergy-S diffractometer equipped with Cu Kα radiation (λ = 1.54178 Å). A HyPix-6000HE detector was employed for data collection. Unit cell parameters were refined using the CrysAlisPro software, and the crystal structures were solved using the intrinsic phasing method with the SHELXT program.^33^ Absorption corrections were applied through the Gaussian method to account for any variations in intensity due to the crystal’s geometry. The final structure models were analyzed and visualized, with tables and structural representations generated using OLEX2 ^34^ for refinement and MERCURY for graphical presentation, as well as full interaction maps (FIMs).^35^ The quality of the data and the refinement process ensure a high degree of accuracy in the structural determination of the complexes. All the data collection and refinement parameters are provided in Table S2 of the Supporting Information.

### 2.5 Theoretical Calculations

To identify the nature and study the interaction occurring between fluoride and *N*-4-bromobenzamide-1,8-naphthylimide, post-Hartree–Fock calculations were performed by using the MP2 method with the resolution of identity approximation (RI-MP2). The 6-311++G** basis set was employed for all of the atoms. Frequency calculations were also carried out at the RI-MP2/6-311++G** level of theory to determine the minimum energies. All calculations were performed using the ORCA 5.0.4 software package.^36^

### 2.6 Luminescence

#### 2.6.1 Interaction of *N*-4-Bromobenzamide-1,8-naphthylimide with Ions by Luminescence

To evaluate the ligand **1** interaction capacity by fluorescence, cations and interfering anions such as Al^3+^, Ca^2+^, Cd^2+^, Cr^2+^, Hg^2+^, Mg^2+^, Na^+^, Ni^2+^, Pb^2+^, Zn^2+^, Cl^–^, Br^–^, Ac^–^, I^–^, F^–^, NO_2_^–^, CN^–^, S^2–^, H_2_PO_4_^–^, HPO_4_^2–^, PO_4_^3–^, CO_3_^2–^, SCN^–^, and SO_4_^2–^ were used. A solution of ligand **1** was prepared at 2 × 10^–5^ mol dm^–3^, and a solution of each ion of interest was prepared at 1 × 10^–2^ mol dm^–3^. All solutions were purged with N_2_ before analysis. The titration was started by adding 1.8 μL of the solution of the ion of interest (1 × 10^–2^ mol dm^–3^), corresponding to a ratio of 1:0.3 (free ligand/ion), and was repeated for the subsequent volumes, varying the aliquots (μL) based on the ratios 1:0.3, 1:0.6, 1:1, 1:1.3, 1:1.6, 1:2, 1:3, 1:4, 1:5, and 1:10.

The quantum yields of ligand **1**, using standard rhodamine (RodG) solution at 1 × 10^–6^ mol dm^–3^ in deaerated acetonitrile, were calculated according to eq 1,^37^ where ϕ*_x_* is the quantum yield of the sample to be determined, ϕ*_s_* is the quantum yield of the standard, *A_x_* is the area under the emission spectrum curve of the standard, Abs*_s_* is the absorption spectrum of the standard, Abs*_x_* is the absorption spectrum of the sample, and *n_s_* ^2^ and *n_x_* ^2^ are the refractive indices of the solvents used to dilute the standard and sample, respectively. Since the same solvents were used, the refractive index is 1.

1 $$\Phi_{x}=\Phi_{s}\left(\frac{A_{x}}{A_{s}}\right)\times \left(\frac{{Abs}_{s}}{{Abs}_{x}}\right)\times \left(\frac{n_{s}^{2}}{n_{x}^{2}}\right)$$

### 2.7 UV–Vis Spectra

#### 2.7.1 Interaction of *N*-4-Bromobenzamide-1,8-naphthylimide with Ions

Titration measurements were performed with cations and interfering anions to evaluate the ligand **1** detection capacity, such as Al^3+^, Ca^2+^, Cd^2+^, Cr^2+^, Hg^2+^, Mg^2+^, Na^+^, Ni^2+^, Pb^2+^, Zn^2+^, Cl^–^, Br^–^, Ac^–^, I^–^, F^–^, NO_2_^–^, CN^–^, S^2–^, H_2_PO_4_^–^, HPO_4_^2–^, PO_4_^3–^, CO_3_^2–^, SCN^–^, and SO_4_^2–^, in the UV–vis region. A solution of ligand **1** at 2 × 10^–5^ mol dm^–3^ and a solution of each ion of interest at 1 × 10^–2^ mol dm^–3^ were prepared. Then, 3 mL of ligand **1** was added to the cuvette, and the first spectrum was recorded. Then, titration was started by adding 1.8 μL of the ion of interest, corresponding to a ratio of 1:0.3 (free ligand/ions), and a new spectrum was recorded. The procedure was repeated for the following volumes, varying the aliquots (μL) based on the ratios of 1:0.3, 1:0.6, 1:1; 1:1.3, 1:1.6, 1:2, 1:3, 1:4, 1:5, and 1:10. All titrations were performed in triplicate, and each measurement’s response was 10 s, and after 5 min, identical spectra (fast equilibrium) were observed.

Figures of merit were made for the fluoride, cyanide, and sulfide anions because the other cations and anions did not respond to this ligand (Table 1). The limit of detection (LOD) and limit of quantification (LOQ) were calculated according to 3 σ/s and 10 σ/s, respectively (where σ and s represent, respectively, the standard deviation of blank solution (*n* = 10) and the slope of the calibration curve).

**Table 1:** Figure of Merits of Anions

anion	λ (nm)	dynamic working range (mol dm^‑3^)	*R*	slope (std error)	LOD (ppm)	LOQ (ppm)
CN^–^	346	6.0 × 10^–5^–1.6 × 10^–4^	0.9890	126.95 (±8.47)	0.1255	0.4184
S^2–^	346	6.0 × 10^–5^–4.0 × 10^–4^	0.9821	40.88 (±2.96)	0.4804	1.600
F^–^	346	2.0 × 10^–5^–2.0 × 10^–4^	0.9938	144.47 (±5.70)	0.081	0.2684

Stoichiometry was determined by the continuous variation method (Job’s plot)^38^ and monitored by electronic spectrophotometry (UV–vis). All experiments were carried out in an acetonitrile (ligand), where the concentrations of ligand **1** and the fluoride ion (water) were kept constant {[Ligand **1**]_t_ + [F^–^]_t_ = M_t_} at 2 × 10^–5^ mol dm^–3^ as the sum of the component concentrations, and the ratio *r* = [F^–^]_t_/{[F^–^]_t_ + [ligand **1**]_t_} varied from 0 to 1.^39,40^

Reversibility studies were performed using a solution of ligand **1** at 2 × 10^–5^ mol dm^–3^ in acetonitrile and alternately added 30 μL of F^–^ and 15 μL of Mg^2+^, both at 1 × 10^–2^ mol dm^–3^ in water_._

### 2.8 Synthesis of Ligand 1

#### 2.8.1 *N*-4-Bromobenzamide-1,8-naphtylimide (**1**)

To 10 mL of ethanol, 0.100 g (5.04 × 10^–4^ mol) of 1,8-naphthalic anhydride and 0.130 g (6.04 × 10^–4^ mol) of 4-bromobenzohydrazide were added. The solution was then heated to 80 °C and stirred vigorously. After 27 h, the transparent solution gradually formed a white solid precipitate (Scheme 1). The reaction was monitored using a dichloromethane/ethyl acetate/methanol solvent mixture (4:5.5:0.5) in Al_2_O_3_, with a final *R*_f_ of 0.66. This solution was transferred to a round-bottom flask to permit more precipitation at room temperature. A white solid was filtered, yielding 0.139 g (69.2%). ^1^H NMR (δ CDCl_3_ 300 MHz): 7.65 ppm (d, 2H, 12-*H*, ^3^ *J* = 8.5 Hz,), 7.81 ppm (dd, 2H, 3-*H*, ^3^ *J* = 7.46 Hz), 7.88 ppm (d, 2H, 11-*H*, ^3^ *J* = 8.5 Hz), 8.30 ppm (dd, 2H, 4-*H*, ^3^ *J* = 8.36 Hz, ^4^ *J* = 0.97 Hz), 8.42 ppm (sl, 1H, 9-*H*), 8.67 ppm (dd, 2H, 2-*H*, ^3^ *J* = 7.46 Hz, ^4^ *J* = 0.97 Hz). ^13^C NMR (75.47 MHz, CDCl_3_) δ: 122.3, 126.6, 127.8, 129.9, 131.4, 134.7, 162.1, 165.4. Elem. Anal. Calc: C_19_H_11_BrN_2_O_3_ C, 57.74; H, 2.81; N, 7.09; found: C, 57.50; H, 2.82 N, 7.02. IR (KBr): 3280 cm^–1^ (*νN*–*H*), 3066 cm^–1^ (*νC*–*H*-*aromatic*), 1685 cm^–1^ (*νC*=*O*-*amide*).

**Scheme 1:**
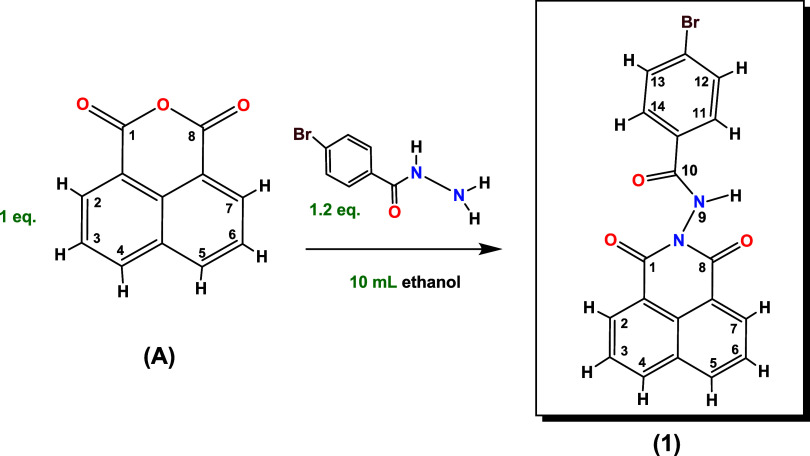
Synthesis of N-4-Bromobenzamide-1,8-naphtyllimide (**1**) in Ethanol

## 3 Results and Discussion

### 3.1 Syntheses and Structural Analysis

The spectrum of 1,8-naphthalic anhydride shows the following signals: 7.85 ppm (dd, 2H, 3-*H*, ^3^ *J* = 7.21 Hz), 8.34 ppm (dd, 2H, 4-*H*, ^3^ *J* = 8.22 Hz, ^4^ *J* = 0.89 Hz), 8.65 ppm (dd, 2H, 2*-H*, ^3^ *J* = 7.22 Hz, ^4^ *J* = 0.89 Hz) (Figure 1). Ligand **1** shows three new signals referring to the protons of the benzene ring and the amide group at 7.65, 7.88, and 8.42 ppm (11-*H*, 12-*H*, and N*H*, respectively) and the signals of the naphthylimide group, 3-*H* at 7.81 ppm; the signal 4-*H* at 8.30 ppm and signal 2-*H* at 8.67 ppm. These changes confirmed that the proposed molecule was obtained.

**Figure 1:**
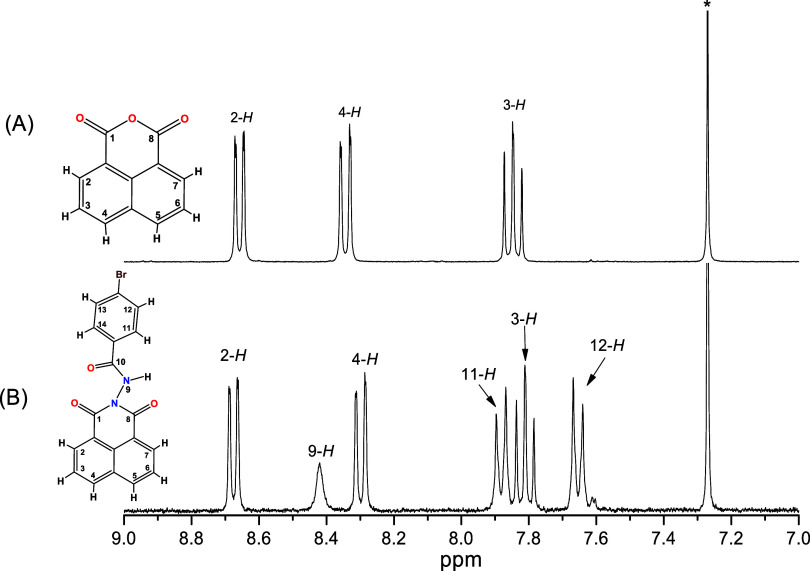
^1^H NMR spectra of 1,8-naphthalic anhydride (A) and ligand **1** (B) in the range of 9.0 to 7.0 ppm in CDCl_3_ (*).

These proposed assignments were confirmed by two-dimensional COSY and HSBC correlations (Figures S1 and S2 and Table S3 in Supporting Information) covering the main carbon signals of this molecule.

Two-dimensional HMBC correlations (including three-bond correlations and weaker two- and four-bond correlations) (Figure S3) were identified in ligand **1**. The molecule under study consists of two rings: one benzene ring and one naphthylimide ring. Because of their symmetry, both rings contain protons in chemically identical environments, resonating at a single δ value. By analyzing the signals from the carbonyls of the amide group and naphthylimide, it was possible to distinguish the signals from the aromatic rings. Thus, (A) at F2:7.91 ppm (11-*H*) in the hydrogen spectrum and F1:166.4 ppm in the carbon spectrum (C=O_10_), there is a hydrogen coupling with the carbon of the amide group carbonyl to three bonds (^3^J_CH_). In (B) at 8.54 ppm (2-*H*) in the proton spectrum, there is a coupling with the carbon at 162.1 ppm of the imide group (C=O_1_) of the naphthylimide to three bonds (^3^J_CH_). We can observe in (C) the coupling of the benzene ring at F2:7.56 ppm (13-*H*) to three bonds at F1:126.4 ppm with the quaternary carbon linked to the carbonyl carbon (C*q*3). At (C′), there are couplings at F2:7.56 ppm (12-*H*), which are coupled to two bonds at F1:131.71 ppm with the carbon (C11). In (D) at F2:7.91 ppm (11-*H*), there is coupling to three bonds at F1:129.8 ppm with the carbon linked directly to the bromine (C*q*-Br). In (D′), at F2:7.91 ppm (11-*H*), there is coupling with two bonds at F1:129.8 ppm, with the benzene portion’s quaternary carbon (Cq3).

On the naphthylimide ring, in (E), proton coupling was observed at F2:7.73 ppm (3-*H*), with the carbon at F1:122.3 ppm (C*q* **1**) to three bonds. In (E′), there is proton coupling at F2:8.22 ppm (4-*H*) to two bonds at F1:122.3 ppm with the carbon (Cq1). In (E″), the hydrogen is coupled at F2:8.53 ppm (2-*H*) to four bonds at F1:122.3 ppm with the carbon (Cq1). In (E′″), the hydrogen is coupled at F2:7.73 ppm (3-*H*) to two bonds at F1:131.4 ppm with the carbon (*C*5). In (**F**), the proton is coupled at F2:8.23 ppm (4-*H*) with the carbon at F1:133.0 ppm (*C5*). In (**G**), at F2:8.23 ppm (4-*H*), proton coupling occurs at a quaternary carbon at F1:127.8 ppm (C*q* **2**), and with (*H*) a carbon at F1:134.5 ppm (*C*2), all being three-bond couplings. In (**I**), the proton at F2:8.54 ppm (2-*H*) couples with the carbons at F1:131.4 ppm (*C*2) and in (**J**) at 127.8 ppm (C*q* **2**), all being three-bonded. The other signals are shown in Table S4, Supporting Information.

### 3.2 Determination of the Structure of Ligand **1** by Single-Crystal X-ray Diffractometry

The crystal structure of ligand **1** (Figure 2) was determined by X-ray diffraction, revealing that the molecule crystallizes in a triclinic crystal system with space group *P*1̅. The asymmetric unit comprises one ligand molecule along with a hydration water molecule. Analysis of the molecular conformation shows that the naphthylimide moiety is essentially planar, while the benzene ring is twisted relative to it, creating an angle of 44.77° between the two planes (Figure S4 of the Supporting Information). This confirms the proposed structure of ligand **1**.

**Figure 2:**
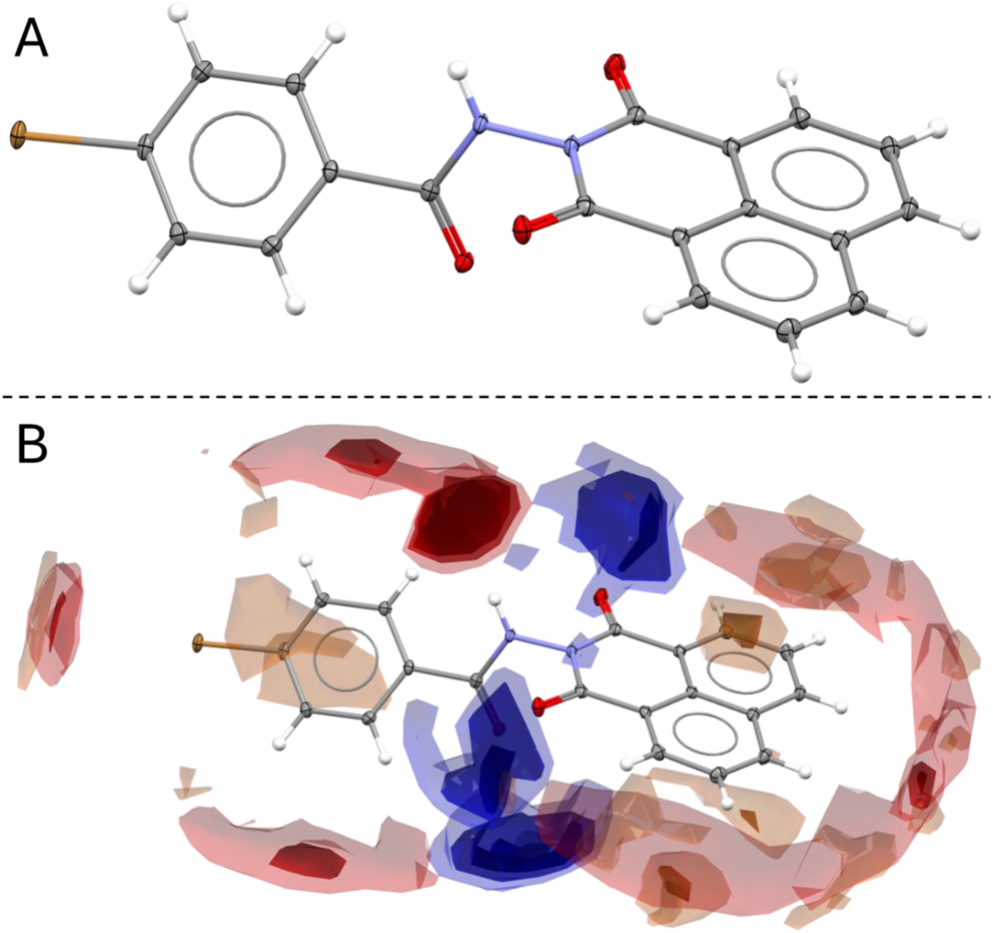
(A) Crystal structure of ligand **1**. The ellipsoids are drawn at 30% probability. (B) Full interaction map (FIM) of ligand **1**. Blue regions around the carbonyl oxygen (C=O) indicate hydrogen bond acceptor sites, red regions around the C–H and N–H groups represent hydrogen bond donor sites, and brown regions around the aromatic rings highlight π–π stacking and C–H···π interactions.

To further investigate the intermolecular interactions governing the crystal packing, a full interaction map (FIM) analysis was performed using Mercury, employing three specific probes: uncharged NH nitrogen, carbonyl oxygen, and aromatic CH carbon. The results reveal a distinct distribution of interaction sites with blue regions surrounding the carbonyl oxygen (C=O) groups, indicating strong hydrogen bond acceptor sites. These interactions suggest the involvement of carbonyl groups in hydrogen bonding, contributing to the stabilization of the crystal structure. The red regions are concentrated around the C–H and N–H groups, marking strong hydrogen bond donor sites, suggesting that these groups participate in hydrogen bonding interactions with electronegative acceptors in the lattice. Brown regions are primarily found around the aromatic rings, highlighting π–π stacking interactions and C–H···π contacts, which play a crucial role in the molecular packing arrangement. These intermolecular forces collectively stabilize the crystal structure, reinforcing the organization of ligand **1** in the solid state.

Notably, hydrogen bonds are observed between the water molecules and C=O groups and between the N–H and water molecules (Figure S5). These interactions play a crucial role in stabilizing crystal packing, thereby contributing to the overall organization and structural integrity of the crystal lattice.

### 3.3 Vibrational Spectroscopy in the Infrared Region (FTIR)

The infrared spectrum of **1** is depicted in Figure S6. The ligand (**1**) stretches differ from those of the starting reagents. Compound (A) has characteristic NH stretches of the secondary amide at 3307 cm^–1^ ^41^ and an associated NH_2_ at 3213 cm^–1^,^42^ and compound (B) has characteristic stretches of the anhydride group at 1770 cm^–1^. All of the ligand (**1**) signals are displaced compared to the starting ligand signals. In the meantime, ligand (**1**) showed bands at 3280 cm^–1^ that were assigned to the (N–H) vibration frequency of the amide groups (R–C=O–NH–R). The vibrations observed at 3066 cm^–1^ are assigned to aromatic C–H in the ligand structure (**1**). The amide group has strong C=O bands appearing in 1685 cm^–1^ in (1), as observed in the secondary amides (1680 to 1630 cm^–1^).^43^

### 3.4 Determination of Acid Dissociation (p*K*_a_) of Ligand **1**

The spectral analysis results conducted during NaOH titration (Figure 3), within the pH range of 5.5 to 9.6, revealed isosbestic points at 242, 253, 336, and 353 nm. This indicates that the ligand and analyte equilibrium was achieved without forming intermediates. The progressive addition of NaOH resulted in absorption bands at 206 and 232 nm, which underwent a bathochromic shift to 212 and 235 nm, respectively, followed by a band at 269 nm. This phenomenon is attributed to the deprotonation of the amide group (R–NH–C=O), which leads to the formation of a monoanion (N–), as observed in Scheme 2. The inset graph in Figure 3 was obtained from log­(*A*_obs_ – *A*_HA_)/(*A*_A_ – *A*_obs_) vs pH, where p*K*_a_ = 9.32 was observed with the zero intersection on the ordinate scale.

**Figure 3:**
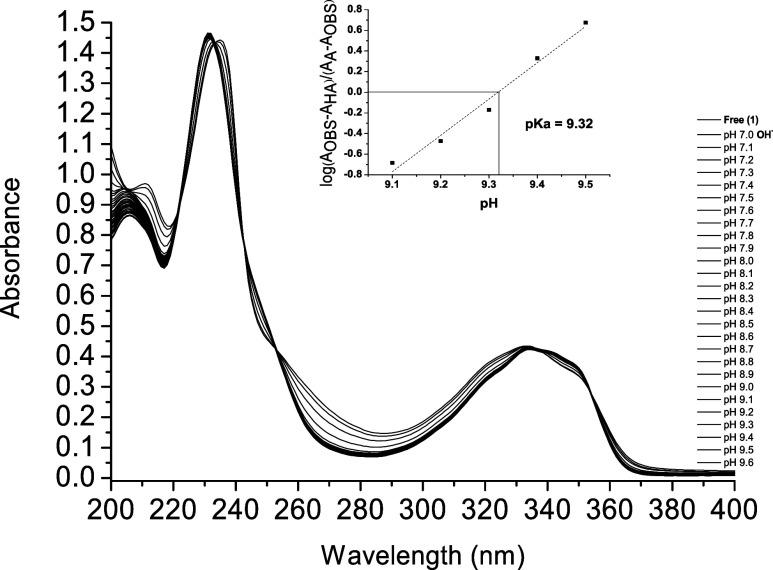
Determination of the p*K*_a_ of the ligand (**1**) at 2 × 10^–5^ mol dm^–3^ in acetonitrile upon addition of NaOH (1 × 10^–2^ mol dm^–3^ in water), on the pH range of 7.0 to 9.8.

**Scheme 2:**
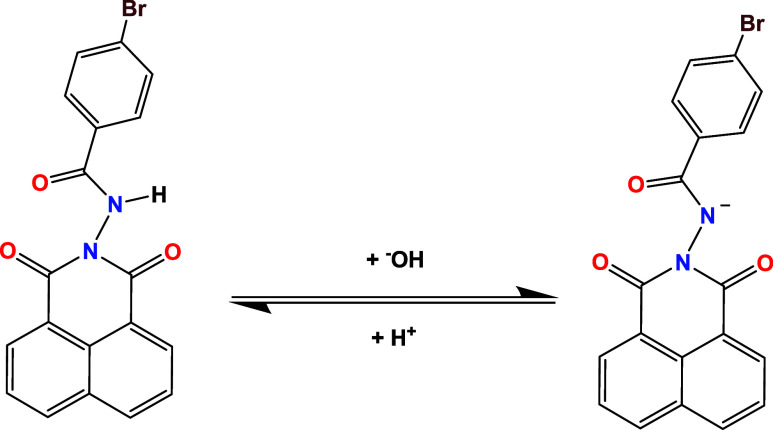
Proposed Mechanism for the Deprotonation Equilibrium of the Amide Group (**1**)

### 3.5 UV–Vis Spectral Studies of Ligand **1** within Fluoride

As described in the Section 2 (Section 2.7), several ions were tested with ligand **1**, including cyanide and sulfide, which showed a poor response with a higher error. It was also calculated and observed that, at the final total concentration of added anions, 2 × 10^–4^ mol dm^–3^, the pH response of the CN^–^ and S^2–^ anions (pH > 9.75) was greater than the p*K*_a_ of the ligand since they are salts that undergo hydrolysis (they are salts of strong bases with weak acids). The calculation of the pH of the solution with fluoride gave pH = 6.75, showing that at this concentration, there is no hydrolysis effect, and the result of the chemosensor is not altered. Thus, fluoride has good sensitivity and a wide dynamic linear working range, in addition to a better *R*, as described in the figures of merit in Table 1. Due to the interesting response to the fluoride anion and practically no interference from other anions, the experiments with fluoride are presented below.

In the spectra of ligand **1**, isosbestic peaks at about 240, 256, 329, and 354 nm were observed with the addition of F^–^ ions (Figure 4). This effect indicates equilibrium between the system’s ligand and analyte. A slight bathochromic shift from 232 to 235 nm was observed, followed by a decrease in the 248 nm band, an increase in the 269 nm band, and a decrease in the 346 nm band. An association constant of *K*_11_ = 2 × 10^4^ mol dm^–3^ (*R* = 0.95) can be seen in the inset in Figure 4, showing strong stability with fluoride. The stoichiometry was determined by the continuous variation method, and a Job plot was constructed by plotting the absorbance variation (Δabs·*r*) as a function of *r* (Figure S7). Then, a symmetrical bell shape with a maximum at *r* = 0.5 was identified, indicating that the association system has a 1:1 stoichiometry, and no other compounds seem to be present.

**Figure 4:**
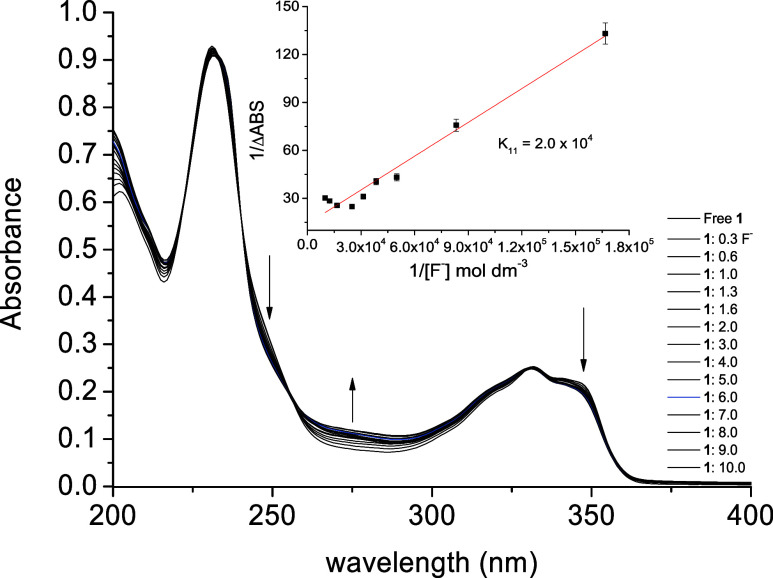
Ligand (**1**) interaction spectrum at 2 × 10^–5^ mol dm^–3^ in acetonitrile and fluoride ions at 1 × 10^–2^ mol dm^–3^ in water. Inset: Binding constant *K*_11_ was determined by the Benesi–Hildebrand method.

This typical interaction should be related to the hydrogen bonding of the N–H group with F^–^, i.e., N–H···F^–^, as shown in the initial proposal in Scheme 3 and described in several works.^2,20–22^ A LOD of 0.081 ppm (*R* = 0.9938) and LOQ of 0.2684 ppm were obtained for this ligand. According to the WHO and EPA, the recommended fluoride level in drinking water is approximately 1 mg/L or 1 ppm (parts per million). This level is considered ideal for preventing tooth decay, while levels above 1.5 mg/L can cause dental fluorosis. Ligand **1** has a low LOD (0.081 ppm and LOQ = 0.2684 ppm), which suggests that the ligand may be suitable for determining environmental fluoride, as this concentration is below that permitted by CONAMA^44^ in aquatic bodies (1.4 ppm).

**Scheme 3:**
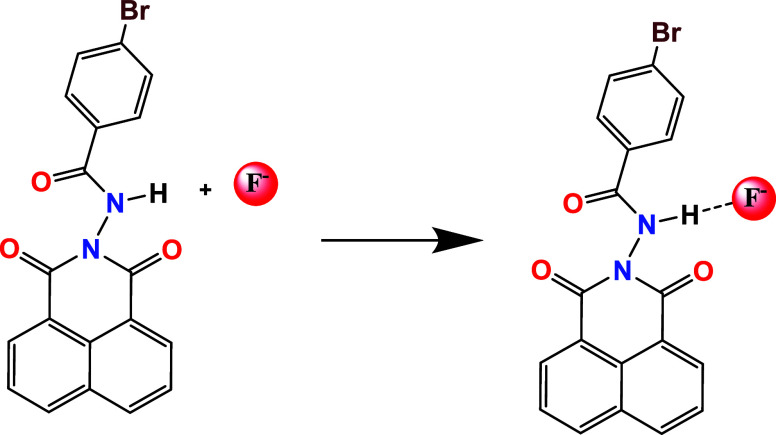
Initial Proposal of the Interaction of Ligand (**1**) with Fluoride Ions

In order to elucidate the reversibility of ligand **1** with F^–^, the process was depicted by UV–vis spectra at 346 nm in acetonitrile. This process involves an initial interaction with fluoride, followed by the addition of Mg^2+^ to capture fluoride. This experiment was similar to one that had been previously conducted.^45^ The zigzag profile shown in Figure S8 represents the decrease of the 346 nm band on fluoride interaction and the increase of the band in the treatment of Mg^2+^ cations. The reversible process could be observed 3 times before saturation. This result demonstrated the reversible process of ligand **1** for the recognition of F^–^.

When comparing the deprotonation spectra, especially when using a strong base such as NaOH, it was observed that the profile of the bands was similar to that of the interaction with fluoride ions. This similarity supports the interaction proposed for the N–H group of the molecule.

However, studies using ^1^H NMR were made to determine the group interaction of this molecule, as described below.

### 3.6 Interaction of *N*-4-Bromobenzamido-1,8-naphthylimide (**1**) with Ions by ^1^H NMR

As previous experiments (UV–vis, Figure 4) showed changes in the ligand bands with the fluoride interaction, the next idea was to determine which group of the molecule the fluoride was interacting with. According to the Section 2.1 (2.3), titration experiments were carried out, resulting in the spectra shown in Figure 5. Surprisingly, the NMR titration did not show an interaction with the NH group, but a probable interaction with the benzene ring through an anion−π interaction. This interaction is known for some planar systems,^46–48^ but has never been observed for rings of systems similar to those in this work. It was observed that in the first spectrum (free ligand), the signal corresponding to the proton donor group (N–H) remained almost unchanged, located at 7.60 ppm (9-*H*). On the other hand, a downfield shift of the signal from 7.88 ppm (11-*H*) to 7.92 ppm was observed. These changes suggest that the benzenic ring interacts with the F^-^ ion via anion−π interactions (see Scheme 4). As we increased the concentration of fluoride ions in the system, the anion−π effect became more evident than hydrogen bonding. This effect is enhanced in aprotic solvents such as CH_3_CN. Furthermore, the presence of the carbonyl group and bromine attached to the benzene ring structure possibly acts as electron density-withdrawing groups, which leaves the benzene ring deficient in electron density, contributing to the interaction of the anion with the π acceptor orbitals of the benzene moiety. As already observed in the single-crystal X-ray crystal (Figure 2), the benzene and naphthylimide rings are approximately 45° apart, and the interaction of the fluoride ion with the benzene ring probably has a minimal electronic change in the naphthylimide rings, resulting in a slight shift of the 2H (8.61 to 8.58 ppm) and 4H (8.44 to 8.41 ppm) signals.

**Figure 5:**
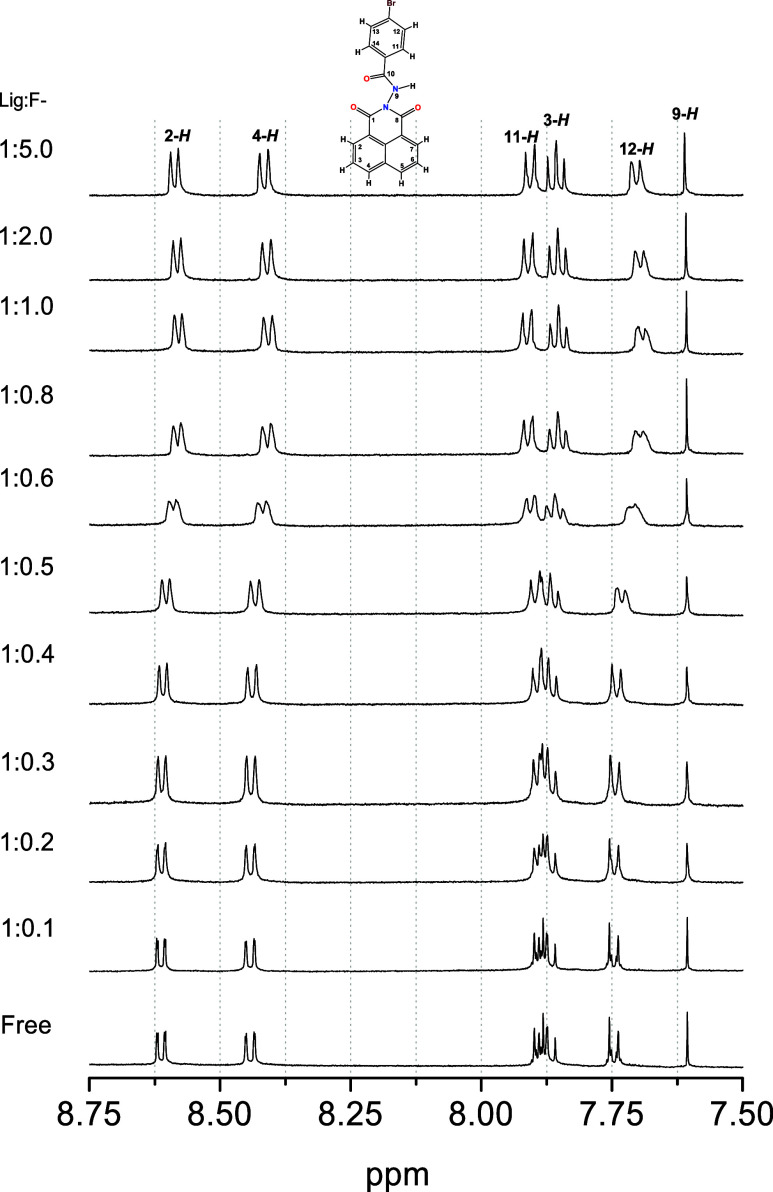
^1^H NMR spectra of **1** (1 × 10^–3^ mol dm^–3^) in CD_3_CN during the addition of F^–^ anions (6.8 × 10^–1^ mol) in D_2_O (1:0.1 to 1:5.0) at 500 MHz (298 K).

**Scheme 4:**
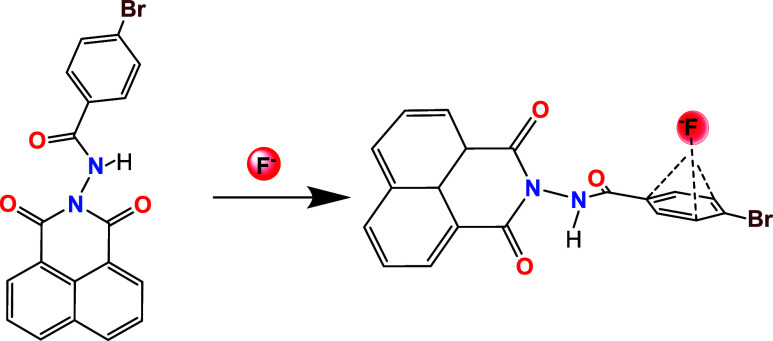
Final Proposed Interaction of the Ligand with Fluoride Ions

### 3.7 Theoretical Investigation of Anion−π Interactions

In association with NMR, a simulation was carried out to provide further evidence related to the anion−π interactions. For calculations involving weak molecular interactions (including anion−π interactions), the basis set superposition error (BSSE) can arise.^49^ This error occurs because, in a quantum calculation describing the interaction between two chemical species, the basis functions of the involved species contribute to the reduction of each other’s energy. As a result, the calculation tends to overestimate the chemical bonding between the species due to basis set effects. Considering the Boys–Bernardi method, this error is corrected as it estimates the energies of the species using the basis set of the hypothetical dimer formed.^50^

The structure calculated with RI-MP2/6-311++G** is shown in Figure 6. The distance between the fluoride ion and the center of the aromatic ring is 2.812 Å. This value is similar to distances calculated in various studies on anion−π interactions involving fluorine and π-electrons in conjugated rings.^44,46,51–55^ Visually, it can be observed that the fluoride ion is located at the center of the ring, which is corroborated by the F–C distances (with an average of 3.136 Å), showing a variance of 0.00042, thus centralizing the F^–^.

**Figure 6:**
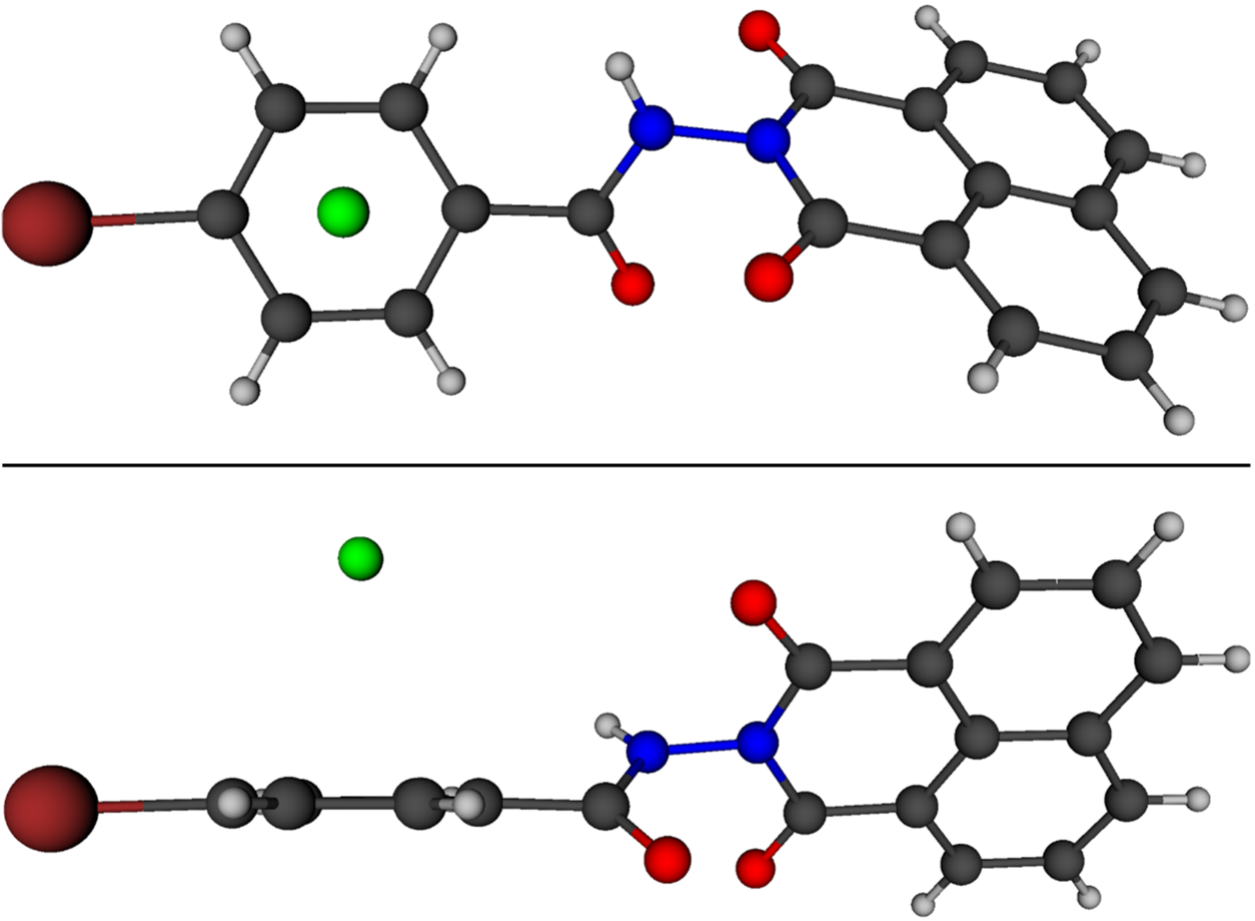
Optimized structure with RI-MP2/6-311++G** using the Boys–Bernardi correction at different angles.

The interaction between F^–^ and the molecule results in an energetic stabilization of −7.53 kcal/mol for the complex, suggesting that the interaction with the aromatic ring is favorable. This energy value is similar to those obtained in studies investigating anion−π interactions involving fluoride and aromatic rings, particularly trifluorobenzene.^51,53^

The molecular orbitals HOMO, HOMO-1, HOMO-2, HOMO-3, and HOMO-4 can be used to study the fluoride ring interaction (Figure 7), demonstrating direct interactions between the species. The HOMO and HOMO-1 orbitals represent out-of-phase interactions between the p orbitals of fluoride and the π orbitals of the ring. In contrast, HOMO-2 shows an exclusive contribution from the p orbitals of fluoride. Finally, the lower-energy orbitals, HOMO-3 and HOMO-4, highlight a subtle in-phase overlap between the orbitals of F^–^ and ring. The molecular orbitals resemble those obtained by Quiñonero et al.^51^ who investigated the interactions between fluorine and aromatic rings.

**Figure 7:**
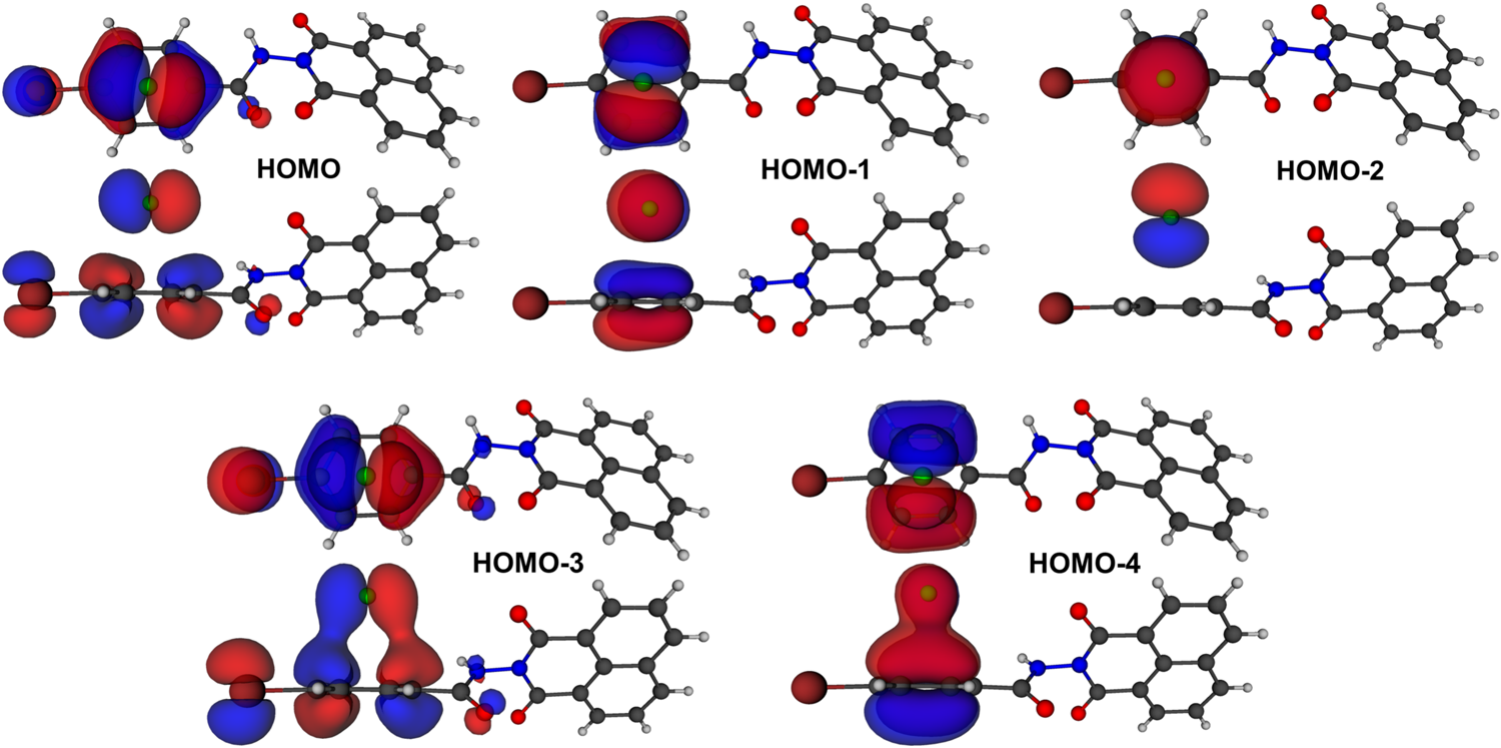
Molecular orbitals with anion−π interactions were calculated with RI-MP2/6-311++G**.

### 3.8 Luminescence Studies of Ligand **1**

The emission spectrum of ligand (**1**) (Figure 8a) presents bands at 381, 401, and 427 nm (λ_exc_ = 346 nm), and the excitation spectrum shows three bands at 333, 343, and 347 nm (λ_em_ = 380 nm), which is a mirror image of the absorption spectrum. In both cases, these bands are assigned to the π → π* transitions of the 1,8-naphthymide ring.^56,57^ The more intense emission band at 381 nm is attributed to the transition from the excited singlet state to the ground singlet state (S_1_ → S_0_).^58–60^ The other bands are assigned to vibrational levels from the excited state to the ground singlet state. A quantum yield of 2.5% was calculated using eq 1 (Section 2.6) for ligand **1**. Quantum yield values were found in the literature for similar compounds,^61,62^ showing that the additional NH group reduces the quantum yield significantly.

**Figure 8:**
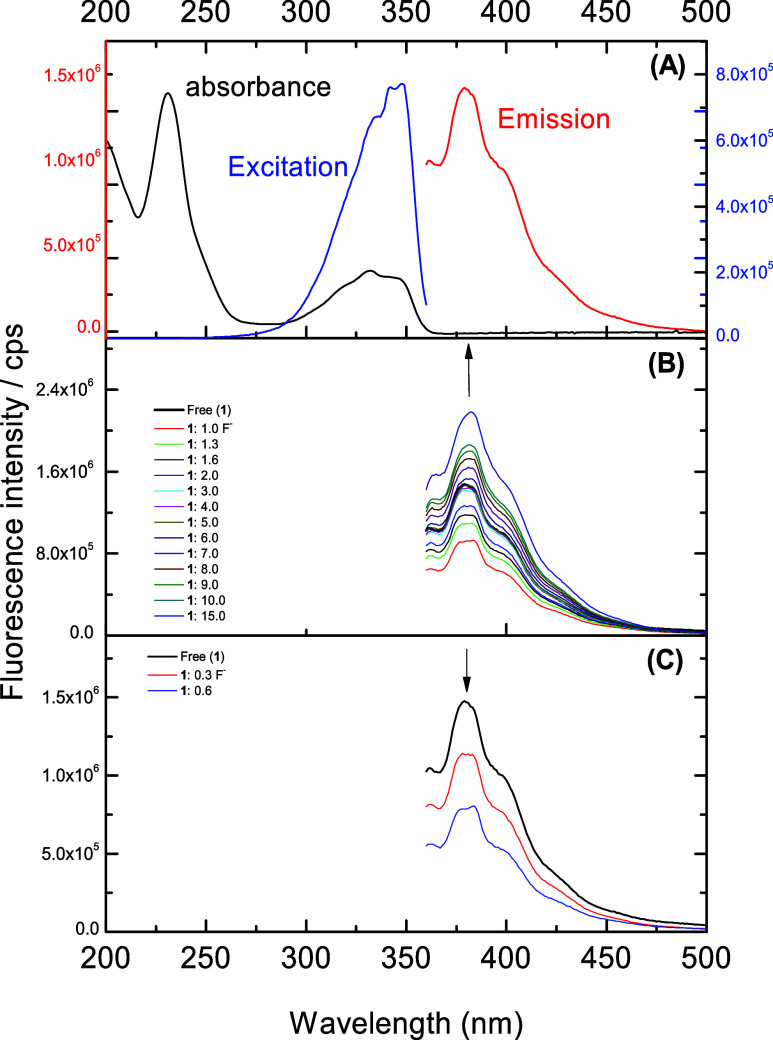
(A) Emission spectrum in red, excitation spectrum in blue, and absorbance spectrum in black. (B, C) Ligand at 2 × 10^–5^ mol dm^–3^ in acetonitrile and F^–^ ions at 1 × 10^–2^ mol dm^–3^ in water.

Similar to the UV–vis results, titration of the ligand with cations and anions did not yield an optimal response, only poor responses for cyanide and sulfide as well as carbonate and phosphate. However, an unexpected response was observed for fluoride, as described below.

An interesting result was observed when the titration of ligand **1** with fluoride ions depicted a suppression of the ligand signals up to 0.6 equiv (Figure 8c). This process may be connected to the interaction with fluoride, suggesting that the emitted energy level (excited state) undergoes a reaction coupled with fluoride, decreasing fluorescence.^63^ However, after 1.0 equiv (Figure 8b), an increase in fluorescence intensity was observed, indicating that this process may be related to the higher stability of the excited state because the molecule assumes a stacking configuration,^64^ which would promote an increase in emission (sensitization).^65^ In fact, this interaction is rather an anion−π electron interaction of the benzene ring with fluoride, as also observed in the NMR and corroborated by theoretical calculations (Figure 7).

A rigorous examination of the free energy and enthalpy of the anion−π interactions, as derived from the experimental fluorescence data, provides unequivocal evidence that validates the hypothesis of a weak interaction. The measurements were conducted at temperatures ranging from 20 to 40 °C, and the values of Δ*G* were determined to be between −2.56 and −3.86 kJ mol^–1^ (−0.612 and −0.923 kcal mol^–1^). These values indicate that the equilibrium is favorable, and the interactions are weak. This observation is primarily attributable to the value of the enthalpy change, Δ*H*, which was determined to be +36.2 kJ/mol (+8.65 kcal/mol) based on a single plot of the Gibbs free energy, Δ*G*, as a function of temperature. This value indicates that the process is primarily an electrostatic interaction (F···π electrons of the ring) rather than a chemical bond, for which the enthalpy values would be negative. The literature shows that in an anion−π system with F^–^,^66^ a positive Δ*H* value indicates an endothermic reorganization of the solvent layer into a less ordered form upon formation of the ligand–fluoride ion interactions.

## 4 Conclusions

In summary, a novel chemosensor, *N*-4-bromobenzamide-1,8-naphthylimide, was synthesized and characterized using a variety of techniques, including ^1^H and ^13^C NMR, IR, UV–vis, elemental analysis, and crystal structure by X-ray diffraction. These techniques confirmed the proposed structure and interactions with fluoride ions. ^1^H NMR titration established that the interaction between the chemosensor and the analyte was of the type involving an anion−π interaction. Furthermore, theoretical calculations provided a basis for the proposed interactions, complementing the experimental results. The chemosensor exhibited a quantum yield of 2.5% and a p*K*_a_ value of 9.32. Furthermore, analysis of the emission process revealed a spatial stacking organization as the concentration of the fluoride ion increased, corroborating the ring organization because of the aforementioned anion−π interaction. This type of interaction for this chemosensor is quite peculiar and could be a promising tool for environmental and analytical applications.

## Supplementary Material





## Notes

2426467 (CIF)
Additional tables, 2D NMR spectra, Job′s plot, and additional figures of the crystal structure of ligand **1** (DOCX)
