# Supplementary material for: Naphthylimide Chemosensor Based on Anion−π Interactions: A Promising Tool for Environmental Monitoring
Source: ACS Omega. 2025 Jul 10;10(28):30684–94. doi: 10.1021/acsomega.5c02699 (PMC12290710; doi:10.1021/acsomega.5c02699)
Supplement: Supplementary file 2 [file ao5c02699_si_002.docx]

**Supporting Information**

*Naphthylimide Chemosensor Based on Anion-π Interaction: A Promising Tool for Environmental Monitoring*

# *Janail Rodrigues da Silva^a^, Vinicius Flores da Silva^a^, Willyan Farias Oliveira^b^, Ricardo Oliveira Freire^b^, João Honorato de Araujo-Neto^c^ and Izilda A. Bagatin^a^**

*^a^Instituto de Ciências Ambientais, Químicas e Farmacêuticas - Depto de Química, Laboratório de Química de Calixarenos, Espectroscopia Molecular e Catálise,*

*Universidade Federal de São Paulo - Rua Prof. Arthur Riedel, 275, CEP 09972-270, Diadema - SP, Brazil*

*^b^Pople Computational Chemistry Laboratory, Department of Chemistry, UFS, São Cristóvão - SE, Brazil, 49107-230.*

*^c^Department of Fundamental Chemistry, Institute of Chemistry, University of São Paulo. 05508-000 São Paulo, SP, Brazil*

**Table** **S1** – Volume of sodium hydroxide added to the solution and corresponding pH (apparent).

| Mesure | Volume (μL) | pH |  | Mesure | Volume (μL) | pH |
| --- | --- | --- | --- | --- | --- | --- |
| 1ª | 0.03 | 7.0 |  | 19ª | 1.93 | 8.8 |
| 2ª | 0.04 | 7.1 |  | 20ª | 2.94 | 8.9 |
| 3ª | 0.05 | 7.2 |  | 21ª | 2.43 | 9.0 |
| 4ª | 0.06 | 7.3 |  | 22ª | 3.85 | 9.1 |
| 5ª | 0.07 | 7.4 |  | 23ª | 4.85 | 9.2 |
| 6ª | 0.09 | 7.5 |  | 24ª | 6.11 | 9.3 |
| 7ª | 0.12 | 7.6 |  | 25ª | 7.71 | 9.4 |
| 8ª | 0.15 | 7.7 |  | 26ª | 9.71 | 9.5 |
| 9ª | 0.19 | 7.8 |  | 27ª | 12.2 | 9.6 |
| 10ª | 0.24 | 7.9 |  | 28ª | 15.4 | 9.7 |
| 11ª | 0.30 | 8.0 |  | 29ª | 19.4 | 9.8 |
| 12ª | 0.37 | 8.1 |  | 30ª | 24.4 | 9.9 |
| 13ª | 0.47 | 8.2 |  | 31ª | 30.7 | 10.0 |
| 14ª | 0.60 | 8.3 |  | 32ª | 38.7 | 10.1 |
| 15ª | 0.76 | 8.4 |  | 33ª | 48.7 | 10.2 |
| 16ª | 0.95 | 8.5 |  | 34ª | 61.3 | 10.3 |
| 17ª | 1.20 | 8.6 |  | 35ª | 77.1 | 10.4 |
| 18ª | 1.53 | 8.7 |  | 36ª | 97.1 | 10.5 |

**Table S2** – Crystal data and structure refinement for ligand 1

| CCDC code | 2426467 |
| --- | --- |
| Empirical formula | C_19_H_13_BrN_2_O_4_ |
| Formula weight | 413.22 |
| Temperature/K | 99.99(10) |
| Crystal system | triclinic |
| Space group | P-1 |
| a/Å | 7.07900(10) |
| b/Å | 9.4784(2) |
| c/Å | 12.3446(2) |
| α/° | 97.4480(10) |
| β/° | 90.7050(10) |
| γ/° | 97.9080(10) |
| Volume/Å^3^ | 813.11(2) |
| Z | 2 |
| ρ_calc_g/cm^3^ | 1.688 |
| μ/mm^‑1^ | 3.709 |
| F(000) | 416.0 |
| Crystal size/mm^3^ | 0.209 × 0.127 × 0.056 |
| Radiation | Cu Kα (λ = 1.54184) |
| 2Θ range for data collection/° | 9.504 to 140.14 |
| Index ranges | -8 ≤ h ≤ 8, -11 ≤ k ≤ 11, -14 ≤ l ≤ 15 |
| Reflections collected | 17021 |
| Independent reflections | 3089 [R_int_ = 0.0322, R_sigma_ = 0.0216] |
| Data/restraints/parameters | 3089/0/238 |
| Goodness-of-fit on F^2^ | 1.080 |
| Final R indexes [I>=2σ (I)] | R_1_ = 0.0226, wR_2_ = 0.0562 |
| Final R indexes [all data] | R_1_ = 0.0252, wR_2_ = 0.0572 |
| Largest diff. peak/hole / e Å^-3^ | 0.54/-0.58 |

**Figure S1 -** ^1^H-^1^H COSY NMR spectrum of ligand **1**, on the range 7.0 to 9.0 ppm, in CDCl_3_/DMSO solvent mixture.

**Figure S2-** ^1^H-^13^C HSQC NMR spectrum of ligand **1** on the range 7.0 a 9.0 ppm (^1^H) and 115-150 ppm (^13^C) in CDCl_3_/DMSO solvent mixture.

**Table S3**. ^1^H-^1^H COSY and ^1^H-^13^C HSQC NMR assignment of ligand 1

| ** (δ*C*)  ppm | Assignment ^1^H | Assignment ^13^C |
| --- | --- | --- |
| (E) 8.54 (131.3) | 2-H | C2 |
| (B) 7.73 (126.5) | 3-H | C3 |
| (D) 8.23 (133.9) | 4-H | C4 |
| (C) 7.91 (129.4) | 11-H | C11 |
| (A) 7.56 (131.1) | 12-H | C12 |

**Figure S3 -** ^1^H-^13^C HMBC NMR spectrum of ligand **1** on the range 7.0 a 9.0. (^1^H) and 110-170 ppm (^13^C) in (CDCl_3_/DMSO) solvent mixture.

**Table S4 -** ^1^H-^13^C HMBC NMR assignment of ligand 1

| δ*H*(δ*C*)/ppm | ^n^J_(CH)_ | Assignment ^1^H | Assignment ^13^C |
| --- | --- | --- | --- |
| 8.54 (134.7) | ^2^J_(CH)_ | 2-H | Cq |
| 8.54 (126.4) | ^2^J_(CH)_ | 2-H | C3 |
| 8.23 (122.3) | ^2^J_(CH)_ | 4-H | Cq |
| 8.23 (131.4) | ^2^J_(CH)_ | 4-H | C3 |
| 7.73 (131.4) | ^2^J_(CH)_ | 3-H | C4 |
| 7.73 (131.4) | ^2^J_(CH)_ | 3-H | C2 |
| 7.91 (126.4) | ^2^J_(CH)_ | 12-H | C11 |
| 7.56 (126.4) | ^2^J_(CH)_ | 11-H | C12 |


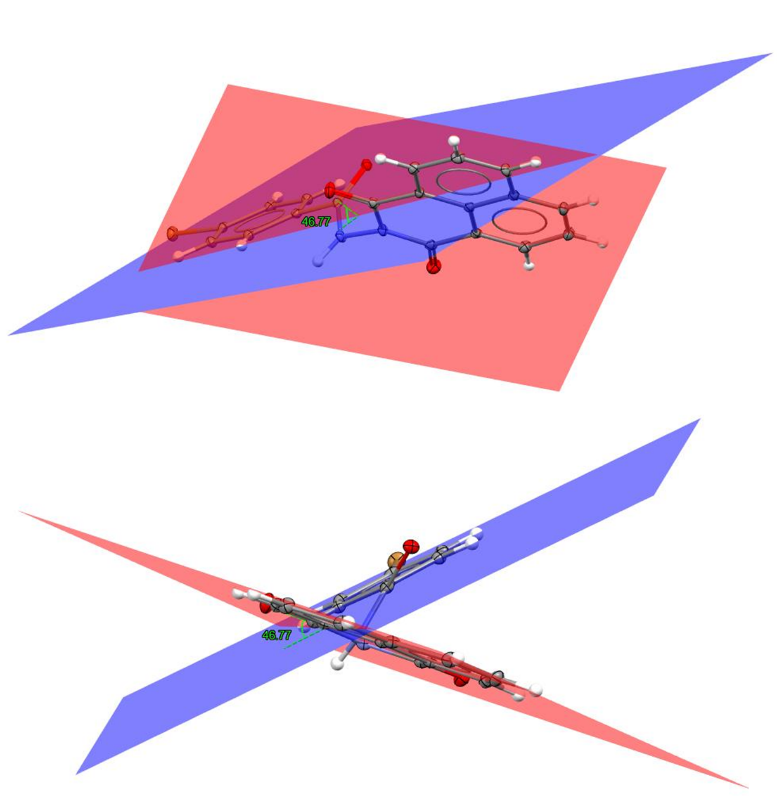


**Figure S4**. The crystal structure of ligand 1 showing the angle between the planes of the naphthylimide moiety and the benzene ring. The ellipsoids are drawn at 30% probability.


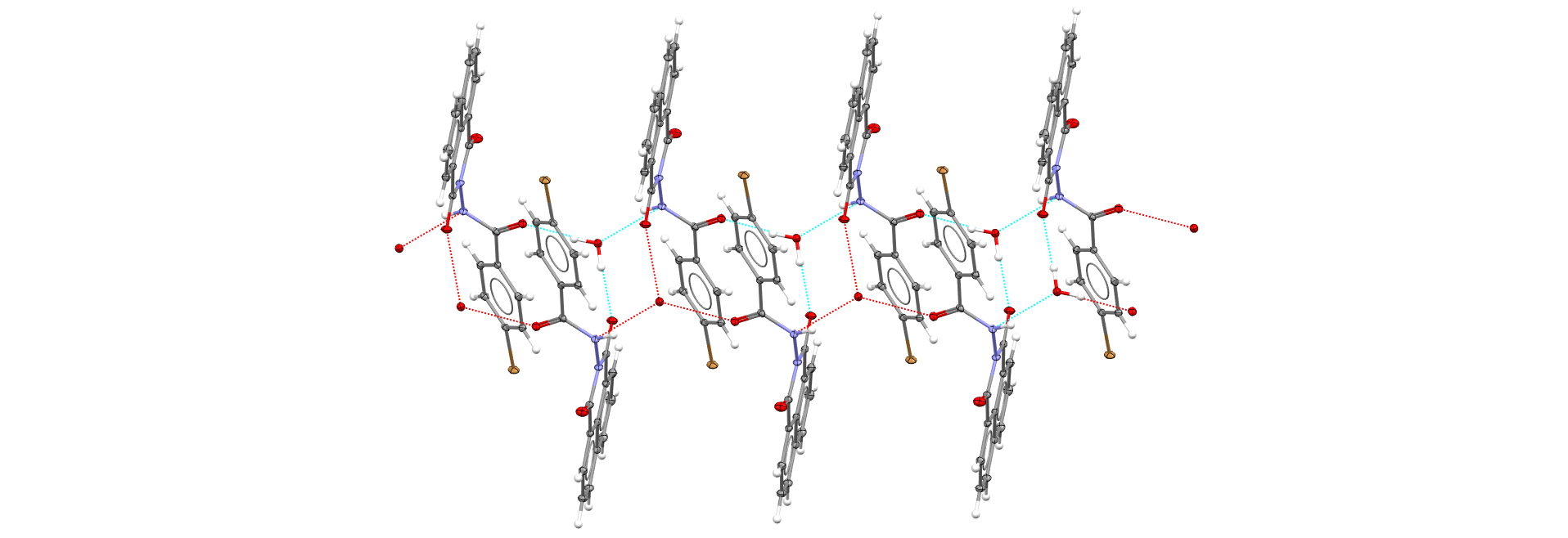


**Figure S5**. Intermolecular interactions in the crystal packing of ligand 1.

**Figure S6 -** FTIR spectrum of the 4-Bromobenzohydrazine (a), 1,8 naphthalic anhydride (b), and ligand **1** (c) on the KBr matrix.

**Figure S7 -** Job Plots of a 1:1 interaction of ligand **1** and fluoride, plotted absorbance variation vs. r against the ratio r=[F^-^]_t_/{[F^-^]_t_+[ligand **1**]_t_}, at an invariant total concentration of 2 × 10^−5^ mol dm^−3^.

**Figure S8 -** UV-Vis spectra reversibility of ligand **1** (2 x 10^-5^ mol dm^-3^) with alternative addition of appropriate uL of F^-^ (1 x 10^-2^ mol dm^-3^) and Mg^2+^ (1 x 10^-2^ mol dm­^-3^).
